# Gastro-esophageal diagnostic workup before bariatric surgery or endoscopic treatment for obesity: position statement of the International Society of Diseases of the Esophagus

**DOI:** 10.1093/dote/doae006

**Published:** 2024-01-27

**Authors:** Pierfrancesco Visaggi, Matteo Ghisa, Brigida Barberio, Philip W Chiu, Ryu Ishihara, Geoffrey P Kohn, Sergey Morozov, Sarah K Thompson, Ian Wong, Cesare Hassan, Edoardo Vincenzo Savarino

**Affiliations:** Gastroenterology Unit, Department of Translational Research and New Technologies in Medicine and Surgery, University of Pisa, Pisa, Italy; Digestive Endoscopy Unit, Pisa University Hospital, Pisa, Italy; Digestive Endoscopy Unit, Department of Gastroenterology, Padua University Hospital, Padua, Italy; Division of Gastroenterology, Department of Surgery, Oncology and Gastroenterology, University of Padua, Padua, Italy; Department of Surgery, School of Clinical Medicine, LKS Faculty of Medicine, The University of Hong Kong, Hong Kong SAR, China; Department of Gastrointestinal Oncology, Osaka International Cancer Institute, Osaka, Japan; Department of Surgery, Monash University Eastern Health Clinical School, Melbourne, Australia; Melbourne Upper GI Surgical Group, c/o Cabrini Hospital, Malvern, Australia; Department of Gastroenterology, Hepatology and Nutrition, Federal Research Center of Nutrition, Biotechnology and Food Safety, Moscow, Russia; College of Medicine & Public Health, Flinders University, Bedford Park, Australia; Department of Surgery, School of Clinical Medicine, LKS Faculty of Medicine, The University of Hong Kong, Hong Kong SAR, China; Department of Biomedical Sciences, Humanitas University, Milan, Italy; Department of Biomedical Sciences, Endoscopy Unit, IRCCS Humanitas Clinical and Research Center, Milan, Italy; Division of Gastroenterology, Department of Surgery, Oncology and Gastroenterology, University of Padua, Padua, Italy; Gastroenterology Unit, Azienda Ospedale Università of Padua, Padua, Italy

**Keywords:** bariatric endoscopy, bariatric surgery, gastro-esophageal workup, ISDE, position statement

## Abstract

Obesity is a chronic and multifactorial condition characterized by abnormal weight gain due to excessive adipose tissue accumulation that represents a growing worldwide challenge for public health. In addition, obese patients have an increased risk of hiatal hernia, esophageal, and gastric dysfunction, as well as gastroesophageal reflux disease, which has a prevalence over 40% in those seeking endoscopic or surgical intervention. Surgery has been demonstrated to be the most effective treatment for severe obesity in terms of long-term weight loss, comorbidities, and quality of life improvements and overall mortality decrease. The recent emergence of bariatric endoscopic techniques promises less invasive, more cost-effective, and reproducible approaches to the treatment of obesity. With the endorsement of the International Society for Diseases of the Esophagus, we started a Delphi process to develop consensus statements on the most appropriate diagnostic workup to preoperatively assess gastroesophageal function before bariatric surgical or endoscopic interventions. The Consensus Working Group comprised 11 international experts from five countries. The group consisted of gastroenterologists and surgeons with a large expertise with regard to gastroesophageal reflux disease, bariatric surgery and endoscopy, and physiology. Ten statements were selected, on the basis of the agreement level and clinical relevance, which represent an evidence and experience-based consensus of the International Society for Diseases of the Esophagus.

## INTRODUCTION

Obesity—defined as body mass index (BMI) ≥ 30 kg/m^2^ outside Asia and as BMI ≥ 25 kg/m^2^ for Asian individuals—is a chronic and multifactorial condition characterized by abnormal weight gain due to excessive adipose tissue accumulation that represents a worldwide growing challenge for public health.^1^^,^^2^ In Europe, a total of €81 billion has been estimated to be spent per year for bariatric patients’ management.^3^ Obesity requires a multidisciplinary approach to both prevention and treatment.^4^ Surgery has been demonstrated to be the most effective treatment for severe obesity in terms of long-term weight loss, comorbidities, and quality of life (QoL) improvements and overall mortality decrease.^5^ Laparoscopic sleeve gastrectomy (LSG) represents the most common procedure, accounting for 59.4% of the 228,000 annual bariatric surgical procedures performed in the United States.^3^ LSG entails resection of the gastric fundus and greater curvature through a partial vertical gastrectomy, which leads to a gastric tubulization.^6^ Other common surgical procedures include gastric banding (GB), Roux-en-Y gastric bypass (RYGB), mini gastric bypass, biliointestinal bypass, biliopancreatic diversion, and endoscopic balloon placement (EB).^5^^,^^7–11^ Independently from the type of surgery, the final result is effective body weight loss and improvement in QoL and comorbidities. Of note, the recent emergence of bariatric endoscopic techniques promises the possibility of less invasive, more cost-effective, and reproducible approaches to the treatment of obesity.^12^

All of the abovementioned procedures have been associated with varying degrees of gastroesophageal functional impairment, with the most clinically relevant being esophageal motility abnormalities and exacerbation of gastro-esophageal reflux disease (GERD).^10^^,^^13–17^ However, there is currently a lack of guidance on the preoperative assessment by endoscopy, manometry, or reflux monitoring before bariatric surgery. In particular, there is uncertainty with regard to which patients need preoperative assessment of GERD, as well as optional versus mandatory investigations and surgical implications of patients where a hiatal hernia (HH) is diagnosed. Similarly, it is unclear whether a subgroup of patients should undergo reflux monitoring and/or manometry, as well as the implications of these tests on the surgical approach. Third, there is uncertainty on the possible preoperative or postoperative benefit of antireflux medication. This Evidence and experience-based Consensus of the ISDE aims to provide recommendations on the preoperative gastro-esophageal diagnostic workup before bariatric surgery or endoscopic treatment for obesity.

## METHODS

With the endorsement of the International Society for Diseases of the Oesophagus (ISDE), we ran a Delphi process to develop consensus statements on the most appropriate diagnostic workup to assess gastro-esophageal function before bariatric surgical or endoscopic interventions, with a special focus on esophageal conditions. This approach combined the evidence-based medicine, systematic literature reviews, and the use of a voting process and is commonly used for determining consensus when high-quality evidence from randomized controlled trials (RCTs) are lacking.^18^ The principal steps of the process were: (i) selection of an International Consensus Working Group to contribute to the expert panel; (ii) proposal of key clinical questions to develop statements; (iii) systematic literature searches and reviews to identify and synthesize evidence to support each statement; (iv) three rounds of repeated voting on iterations of the statements (with feedback at each round) until consensus voting was reached; and (v) grading of the strength and quality of the evidence and recommendations using simplified GRADE criteria, due to limitations of available evidence.^18^^,^^19^ In this regard, owing to the paucity of high-quality evidence, this is considered a Position Statement and not a Clinical Guideline. In this Position Statement, the grading of recommendation was based on both quality of available evidence and strength of the Delphi agreement. A strong recommendation suggests that the intervention should be offered to most patients most of the time whereas a conditional recommendation suggests that there is either lower quality evidence, the balance between benefits and risks is equivocal and/or important uncertainty about patients’ values and preferences exists.

The Consensus Working Group comprised 11 international experts from 5 Countries (Italy; Hong Kong SAR, China; Japan; Australia; Russia). The group consisted of gastroenterologists and surgeons with large expertise with regards to GERD, bariatric surgery and endoscopy, and physiology. The group was initially asked to develop statements on the gastroesophageal diagnostic workup and to provide clinical questions, structured by population, intervention, comparator, and outcome (PICO). Participants were assigned to panels corresponding to statements and developed pertinent summaries for each statement using the available literature. These summaries were written by the panel members and included all the relevant evidence identified for each statement, making specific reference to any studies that were assessed but which did not contribute additional evidence. The Working Group developed an initial nine statements and prepared and reviewed the evidence to support the statements that were presented to the Consensus Group. The Consensus Group subsequently revised, expanded, and consolidated the statements, ultimately providing 11 statements to start the Delphi process. The Summary Statements were then posted online for voting and feedback to guide refinement. The respondents were asked to choose one of the following for each statement: agree strongly (A+), agree with minor reservation (A), agree with major reservation (A−), disagree with major reservation (D−), disagree with minor reservation (D), or disagree strongly (D+). Participants voted on statements, assessments were made on the basis of the participants’ comments, and judgments were informed of the supporting evidence. We defined consensus as 80% of respondents strongly agree (A+) or agree with minor reservation (A). When agreement was not reached, we rephrased the statement to see if this would provoke stronger agreement. If no strong agreement was reached after at least two rounds of voting, it was eliminated. We electronically collected conflicts of interest declarations at each stage of the voting process. This study is a secondary analysis of published work and did not involve human subjects or interventions; therefore, it did not require ethics committee review. However, the study was overseen by the ISDE and was subject to the review of ISDE’s guidelines committee. This document received the endorsement of the European Society for Diseases of the Esophagus (ESDE).

### Literature search strategy

Keywords identified from the clinical questions were used to construct literature searches in electronic databases. For the systematic literature search, MEDLINE, EMBASE, EMBASE Classic, and the Cochrane Library were searched from inception to 31st July 2023 to identify relevant literature for each PICO question. To identify potentially eligible studies published only in abstract form, conference proceedings (Digestive Disease Week, American College of Gastroenterology, and United European Gastroenterology Week) from 2000 until 31^st^ July 2023 were also searched. There were no language restrictions. We screened titles and abstracts of all citations identified by our search for potential suitability and retrieved those that appeared relevant to examine them in more detail. Foreign language papers were translated. A recursive search of the literature was performed using bibliographies of all relevant studies. The references cited in this manuscript represent only a selection of the articles reviewed in each area and were selected to clarify the discussion.

## RESULTS

**Fig. 1:**
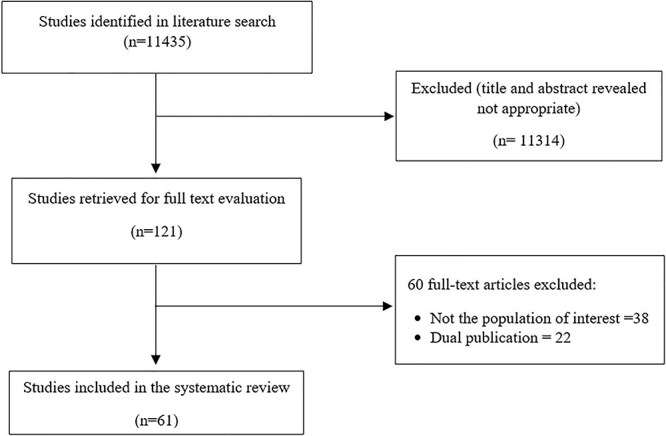
Flow diagram of the literature research.

The literature search yielded 11,435 studies. After screening of title and abstract, 121 were found to be potentially relevant to the study questions. After full-text evaluation, 61 were considered for the preparation of the guideline (Fig. 1). The overall quality of evidence related to all the statements varied from very low to moderate. At the final round of consensus voting, consensus (i.e. agreement ≥80%) was achieved on ten out of 11 statements, while one statement was not accepted (agreement 72.7%). Ten statements were finally selected, on the basis of the agreement level and clinical relevance, which represent an evidence and experience-based consensus of the ISDE on the preoperative gastro-esophageal diagnostic workup before bariatric surgery or endoscopic treatment for obesity (Table 1).

**Table 1:** Summary of statements

**Statement**	**Consensus score**	**Recommendation and quality of evidence**
1. Preoperative EGDS should be considered in all patients planning to undergo bariatric surgery	90.9%	Conditional recommendation—low-quality evidence
2. Preoperative identification and subsequent repair of hiatal hernia larger than 2 cm may improve outcomes in patients undergoing bariatric procedures	100%	Conditional recommendation—low-quality evidence
3a) A contrast esophagogram should not be used for the diagnosis of GERD before bariatric procedures 3b) A contrast esophagogram may be performed to identify the presence of hiatal hernia in case of clinical suspicion	90.9%	Strong recommendation—moderate quality of evidence
4. For patients with typical GERD symptoms, esophageal pH-impedance monitoring may be performed to aid in the choice between sleeve gastrectomy and RYGB	90.9%	Conditional recommendation—low quality evidence
5. For adults with atypical manifestations of GERD, preoperative esophageal pH-impedance monitoring may be performed to aid in the choice between sleeve gastrectomy and RYGB due to the risk of worsening GERD symptoms	90.9%	Conditional recommendation—low-quality evidence
6. High-resolution esophageal manometry should be included in the preoperative evaluation of patients planning to undergo bariatric procedures in case of suspicion of esophageal dysmotility	90.9%	Conditional recommendation—very-low quality evidence
7. No recommendation can be made on the screening for *Helicobacter pylori* infection in patients planning bariatric procedures based on currently available evidence	81.8%	No recommendation
8. Sleeve gastrectomy is not recommended for patients with silent GERD	90.9%	Strong recommendation—low-quality evidence
9a) Postoperative EGDS should be performed after bariatric surgery in case of de-novo upper gastrointestinal symptoms 9b) Postoperative EGDS should be performed within 5 years from bariatric surgery to rule out Barrett’s esophagus	100%	Conditional recommendation—very-low quality evidence
10. We recommend PPI use in the postoperative management of patients undergoing gastric bypass or biliopancreatic diversion for the prophylaxis of marginal ulcers	81.8%	Conditional recommendation—very-low quality evidence

## STATEMENTS

1. **Preoperative esophagogastroduodenoscopy should be considered in all patients planning to undergo bariatric surgery.**

Agree: 90.9% [D + (0%); D (9.1%); D− (0%); A− (0%); A (9.1%); A + (81.8%)].

*Conditional Recommendation—Low Quality Evidence*

**Summary of evidence:**

Upper endoscopy is usually performed to exclude organic disease from the upper gastrointestinal tract or to identify benign diseases requiring medical or other treatments. As to the occurrence of esophageal mucosal injuries in patients undergoing bariatric surgery, in a meta-analysis on 594 patients, an association has been demonstrated between obesity and reflux esophagitis (odds ratio, OR 2.23, 95% CI: 1.59–3.11, *P* = 0.000), erosive gastritis, gastric ulcers (benign and malignant), and duodenal ulcers (OR 1.40, 95% CI: 1.14–1.72, *P* = 0.001).^19^ In another meta-analysis, the pooled adjusted OR of esophageal adenocarcinoma for BMI of 25 kg/m^2^ or greater was 2.02 (95% CI: 1.534–2.669; *P* = 0.001), and there was a trend toward a dose–response relationship with an increase in the pooled OR for BMI 25–30 kg/m^2^ and BMI > 30 kg/m^2^ of 1.52 (95% CI: 1.147–2.009; *P* = 0.004) and 2.78 (95% CI: 1.850–4.164; *P* = 0.001).^20^ Additionally, a meta-analysis on 13,434 patients undergoing endoscopy prior to bariatric surgery found a prevalence of Barrett’s esophagus of 0.9% (95% CI: 0.7%–1.3%); *P* < 0.001; *I*^2^ = 58%).^21^

Four different meta-analyses investigated the impact of preoperative esophagogastroduodenoscopy (EGDS) on the medical and surgical management of bariatric patients. A change in surgical management of candidates for bariatric surgery following EGDS has been reported in the range of 3.9% (95% CI: 3%–4.8%) to 20.6% (95% CI: 14.5%–28.2%).^22–25^ However, reported management modifications were highly heterogeneous and ranged from 0% to 90.2% in single studies. Reasons for change in management (defined as delay, change, or cancelation of the procedure) included repair of HH, GERD, esophagitis, Barrett’s esophagus, bezoar, Zenker’s or esophageal diverticula, gastritis, peptic ulcer disease, and premalignant or malignant lesions. In two meta-analyses, preoperative EGDS provided evidence of an absolute contraindication to bariatric surgery in 0.4%–0.8% due to esophageal cancer or varices.^22^^,^^24^ With regard to change in medical management, a meta-analysis on 5140 patients reported a proportion of 27.5% (95% CI: 20.2–34.8; *I*^2^ = 98.6%) preoperative EGDS leading to management modifications. The proportion of medical management changes ranged from 0% to 70.2% in single studies and was mainly due to *Helicobacter pylori* (HP) eradication (76.4%) and medical therapy for gastritis or GERD (23.5%).^22^ One meta-analysis investigated the yield of preoperative EGDS in patients reporting upper gastrointestinal symptoms versus asymptomatic individuals and found that although the proportion of patients with reflux symptoms and gastric pathology was 65%, asymptomatic patients had abnormal findings in 34.1% of cases. However, the proportions of pathologies resulting in a change in management were not stratified by symptoms and meta-analysis of symptom impact was not performed.^25^

In summary, due to the frequency of abnormal findings, preoperative EGDS should be considered as it may lead to a change in the medical and/or surgical management in a proportion of both symptomatic and asymptomatic bariatric patients.

2. **Identification and subsequent repair of HH larger than 2 cm may improve outcomes in patients undergoing bariatric procedures.**

Agree: 100% [D + (0%); D (0%); D− (0%); A− (0%); A (0%); A + (100%)].

*Conditional Recommendation—Low Quality Evidence.*

**Summary of evidence:**

A HH is a medical condition in which abdominal organs, most commonly the stomach, herniate through the esophageal hiatus into the mediastinum and impair the functioning of the gastro-esophageal junction (EGJ), predisposing to GERD.^26^ Of note, HH may be present in nearly 40% of morbidly obese patients.^27^ Whether HH repair (HHR) could improve postoperative outcomes in patients undergoing bariatric procedures is controversial. A recent meta-analysis investigated the effects of concomitant LSG and HHR in patients with GERD who were scheduled for LSG.^28^ From the pooled analysis of 18 randomized (*n* = 1) and nonrandomized (*n* = 17) studies including 937 patients, the authors found that concomitant LSG and HHR had a positive effect on weight loss, erosive esophagitis, and improvement of GERD symptoms. Accordingly, patients treated with LSG + HHR showed a reduction of GERD symptoms (OR = 0.20; 95% CI: 0.10–0.41; *P* < 0.00001) and erosive esophagitis (OR = 0.12; 95% CI: 0.05–0.26, *P* < 0.001) postoperatively. Overall, 68% of patients experienced GERD remission (95% CI: 55–80.9%), 12% de novo GERD (95% CI: 8–16%), and 11% had HH recurrence (95% CI: 4–19%) following LSG + HHR. In addition, when comparing LSG + HHR and LSG without HHR in 265 and 647 patients, respectively, the OR for remission of GERD symptoms was significantly higher in patients undergoing LSG + HHR compared to LSG without HHR (OR = 2.97; 95% CI: 1.78–4.95, *P* < 0.0001). However, there was no significant difference in terms of *de novo* GERD incidence between the two groups. In contrast, another meta-analysis on 838 patients found that although HHR significantly prolonged the procedure time of LSG, there was no differences in terms of GERD improvement in patients undergoing HHR + LSG or LSG alone.^29^ In a RCT,^30^ 100 obese patients with HH who were scheduled for bariatric surgery were randomized to LSG with crural repair or LSG alone. The authors found that LSG with or without HHR had comparable outcomes in terms of postoperative reflux symptoms. Similarly, in a retrospective study on 35 patients, there was no significant difference in terms of reduction of GERD symptoms in patients with preoperative GERD who underwent LSG + HHR compared to LSG alone.^31^ Although HHR + LSG showed positive postoperative outcomes in patients undergoing bariatric surgery, evidence on a possible superiority of LSG + HHR compared to LSG alone is contradictory.^28^^,^^29^ In this regard, most studies were nonrandomized and of low to moderate quality. Accordingly, further randomized studies are needed to increase the quality of available evidence. However, current evidence suggests the need of identifying a HH and treating it accordingly.

3.
  a) **A contrast esophagogram should not be used for the diagnosis of GERD before bariatric procedures**
  b) **A contrast esophagogram may be performed to identify the presence of HH in case of clinical suspicion**

Agree: 90.9% [D + (0%); D (0%); D− (0%); A− (9.1%); A (36.4%); A + (54.5%)].

*Strong recommendation—Moderate quality of evidence*

**Summary of evidence:**

Barium esophagogram (BE) is a radiological investigation in which X-ray films of the esophagus are taken following the ingestion low-density barium sulfate. The test can be used for the assessment of the anatomy and functioning of the esophagus.^32^^,^^33^ Of note, BE has poor sensitivity and specificity for the diagnosis of clinically relevant GERD when compared with ambulatory reflux monitoring (ARM).^34^^,^^35^ Accordingly, BE should not be used for the diagnosis of GERD. In contrast, BE is valuable for the preoperative identification of HH or structural abnormalities of the esophagus. A recent study compared the accuracy of BE and high-resolution esophageal manometry (HRM) for the diagnosis of HH, using as reference the surgical in vivo measurement.^26^ Although HRM was the most accurate test for the identification of HH (specificity 91.5% and sensitivity 94.3%), the performance of BE was similar to HRM, with specificity of 97.9% and sensitivity of 69.8%. Accordingly, BE could be a valuable tool for the identification of HH in the preoperative diagnostic workup of patients planned for bariatric surgery.

4. **For patients with typical GERD symptoms, esophageal pH-impedance monitoring may be performed to aid in the choice between sleeve gastrectomy and**  **RYGB.**

Agree: 90.9% [D + (9.1%); D (0%); D− (0%); A− (0%); A (27.3%); A + (63.6%)].

*Conditional Recommendation—Low Quality Evidence*

5. **For adults with atypical manifestations of GERD, preoperative esophageal pH-impedance monitoring may be performed to aid in the choice between sleeve gastrectomy and**  **RYGB**  **due to the risk of worsening GERD symptoms.**

Agree: 90.9% [D + (0%); D (0%); D− (0%); A− (9.1%); A (18.2%); A + (72.7%)].

*Conditional Recommendation—Low Quality Evidence.*

**Summary of evidence (statements 4 and 5)**:

Patients with BMI of 25–30 kg/m^2^ and BMI greater than 30 kg/m^2^ have a pooled adjusted OR for GERD symptoms of 1.43 (95% CI: 1.158–1.774) and 1.94 (95% CI: 1.468–2.566), respectively, showing a dose–response relationship between BMI and GERD symptoms.^20^ However, GERD symptoms may be absent in case of pathological gastroesophageal reflux and do not always indicate the presence of abnormal acid exposure.^36^ In this regard, ARM is the gold standard diagnostic test to obtain confirmatory evidence of GERD.^37–39^ ARM includes catheter-based or wireless pH-monitoring devices and catheter-based impedance and pH-monitoring devices.^40^ In particular, 24-hour multichannel intraluminal pH-impedance monitoring provides the most comprehensive assessment of gastro-esophageal reflux events to detect acidic (pH < 4), weakly acidic (pH 4–7), and alkaline reflux events (pH > 7), which would not be assessed without impedance monitoring,^39^^,^^41^^,^^42^ as well as adjunctive metrics that support a diagnosis of GERD in the case of diagnostic uncertainty.^37^^,^^38^^,^^43^

Silent GERD, defined as objective evidence of GERD on ARM in the absence of symptoms, may be present in asymptomatic patients planning bariatric surgery,^44^ and a meta-analysis based on results from 10,718 patients undergoing LSG found a postoperative 19% increase in GERD prevalence.^45^ Accordingly, the ISDE panel addressed the question regarding preoperative reflux monitoring in candidates for bariatric interventions.

A recent systematic review included 12 studies on 547 patients assessing ambulatory pH-monitoring results before and after bariatric procedures.^46^ In eight of the included studies, the authors observed an increase in DeMeester Score (DMS) and/or total acid exposure time (AET) following LSG, a decrease in DMS and/or AET after LSG in two studies, an increase of AET and total number of refluxes following LSG and GB but not after EB, RYGB, mini gastric bypass, biliointestinal bypass, or biliopancreatic diversion in one study. Finally, one study reported an increase of AET after LSG, but no difference after GB or EB. *De novo* GERD rate ranged from 17.8 to 69%.^46^ Another systematic review with meta-analysis investigated GERD outcomes following LSG or RYGB based on pH/pH-impedance monitoring in 498 and 347 patients, respectively.^14^ In nine studies, DMS, AET, and number of acid refluxes significantly decreased following RYGB. In contrast, in 14 studies, total and recumbent AET, and total number of reflux episodes and nonacid reflux episodes significantly increased following LSG. In this regard, two recent prospective non-RCTs found that preoperative DMS and management with LSG (OR 12.3, 95%CI: 2.9–52.5) could predict the development of erosive esophagitis and postoperative GERD at 12 months after surgery, respectively.^15^^,^^47^ In a meta-analysis on GERD outcomes after GB in 129 patients, the percentage of patients with pathological reflux based on pH monitoring decreased in five studies, although the mean total reflux time increased in two studies. *De novo* GERD after GB ranged from 14.3 to 30.1%.^9^ In this regard, a recent meta-analysis found that GERD was the cause of 30.4% of conversions from LSG to RYGB, resulting in the resolution of symptoms in 91.3% of patients at 24-month follow-up.^48^ Accordingly, severe postoperative GERD required conversion from LSG to RYGB in 9% of patients included in a recent RCT.^8^ Finally, a prospective study on 222 patients investigating postoperative outcomes of silent GERD (defined as objective evidence of GERD at endoscopy or pH monitoring in absence of symptoms) found that 25% of patients planning bariatric surgery had silent GERD and, of these, 66% became symptomatic following LSG. Additionally, silent GERD accounted for 17% of symptomatic *de novo* GERD.^49^

In conclusion, outcomes of pre-existing or *de novo* GERD following bariatric procedures are inconsistent and have wide heterogeneity across different studies and interventions. However, although of low quality, evidence regarding GERD outcomes indicate that pre-operative ambulatory pH-monitoring may help to guide the management of patients complaining of both typical and/or atypical reflux-like symptoms, potentially away from LSG.

6. **High-resolution esophageal manometry should be included in the preoperative evaluation of patients planning to undergo bariatric procedures in case of suspicion of esophageal dysmotility.**

Agree: 90.9% [D + (0%); D (9.1%); D− (0%); A− (0%); A (27.3%); A + (63.6%)].

*Conditional Recommendation—Very low quality evidence*

**Summary of evidence:**

HRM is a catheter-based diagnostic test that facilitates evaluation of esophageal function and motility,^32^^,^^50–52^ and the diagnosis of motor disorders of the esophagus according to an international consensus classification, known as Chicago classification, now at its fourth iteration.^33^^,^^50^ In this regard, the prevalence of esophageal motility disorders is estimated to be high in several studies, both in symptomatic and asymptomatic obese patients.^53^ Bariatric procedures can induce changes in esophageal motility that are *de novo* or an aggravation of previously existing conditions.^54^^,^^55^ Accordingly, pre-operative assessment of esophageal motility may be relevant in the setting of bariatric procedures. A recent meta-analysis investigated esophageal manometric changes in 492 and 417 patients following LSG and RYGB, respectively.^14^ Overall, lower esophageal sphincter (LES) resting pressure and esophageal body contractile vigor were significantly reduced after LSG but remained unchanged following RYGB. Both LSG and RYGB were associated with an increased risk of ineffective esophageal motility (IEM) postoperatively (risk ratio = 2.82, 95% CI 1.34–5.98; *I*^2^ = 50% for LSG and 2.41; 95% CI 1.38–4.20; *I*^2^ = 12% for RYGB). Similarly, in another systematic review on 402 patients undergoing esophageal manometry before and after bariatric procedures, esophageal vigor decreased in most patients, causing IEM in some patients postoperatively.^46^ In a large-cohort retrospective study,^55^ achalasia or post-obesity surgery esophageal dysfunction (POSED; an ‘achalasia-like’ picture characterized by aperistalsis and increased intragastric pressure) were significantly more frequent in operated compared to nonoperated obese patients. Increasing time since surgery was an independent risk factor for both achalasia and POSED (*P* < 0.05). Another systematic review of 214 patients reported esophageal manometry findings before and after GB.^9^ Out of five studies investigating esophageal dysmotility after GB, four found postoperative impaired peristalsis, while one did not find significant alterations. LES pressure was increased after GB in five studies and was non-significantly decreased in one study.

Although of low quality, available evidence suggests that bariatric procedures may increase the risk of motility disorders development. Accordingly, preoperative esophageal manometry could be considered in the diagnostic work-up of patients planning bariatric surgery or endoscopy for the initial assessment of esophageal motility, but further studies are needed to investigate clinical outcomes of patients who have dysmotility following bariatric procedures.

7. **No recommendation can be made on the screening for *Helicobacter pylori* infection in patients planning bariatric procedures based on currently available evidence.**

Agree: 81.8% [D + (9.1%); D (0%); D− (0%); A− (9.1%); A (45.4%); A + (36.4%)].

No Recommendation.

**Summary of evidence:**

Prevalence estimates of HP infection in obese patients undergoing bariatric procedures are heterogeneous, with meta-analytic studies showing prevalence rates ranging from 0.13% to 49% in both retrospective and prospective studies.^56^^,^^57^ Similarly, the impact of HP infection on surgical outcomes is unclear. In a meta-analysis on 255,435 patients undergoing bariatric procedures, Mocanu et al.^57^ found that HP infection was associated with a 10-fold increase in marginal ulcers formation following RYGB compared to HP-negative patients. However, the rates of bleeding, leak, hospital length of stay, and weight loss were comparable between HP-positive and HP-negative patients. Similarly, another meta-analysis by Smelt et al.^56^ found that there was no difference in the incidence of postoperative complications in HP-positive patients undergoing pre-operative HP eradication therapy compared to HP-negative patients (OR = 0.5 [95% CI: 0.139–1.977], *P* = 0.340). Of note, the results of these meta-analyses are tempered by the high heterogeneity present in all of the analyses. Similarly, other recent cohort studies conducted in 100 patients undergoing LSG found that there was no difference between HP-positive and HP-negative patients in terms of surgical site infection, bleeding, or leakage.^58^

Further prospective and methodologically valuable studies are needed to clarify whether obese patients planning bariatric procedures should undergo routine preoperative screening and eradication of HP infection to prevent postoperative complications.

8. **Sleeve gastrectomy is not recommended for patients with silent GERD.**

Agree: 90.9% [D + (0%); D (9.1%); D− (0%); A− (0%); A (27.3%); A + (63.6%)].

Strong recommendation—Low quality evidence

**Summary of evidence:**

Sleeve gastrectomy has been associated with GERD. A meta-analysis RCTs addressed the outcomes of RYGB and LSG.^59^ Of five RCTs, four compared RYGB and LSG^8^^,^^60–62^ and the other one compared LSG and GB.^63^ In studies comparing RYGB and LSG, worsening of GERD and conversion to another surgery were more frequent in the LSG group.^8^^,^^60^^,^^61^ In a study that assessed QoL using a GERD questionnaire, the LSG group scored worse than the RYGB group.^62^ In a study that compared LSG and GB,^63^ weight loss and lack of hunger after 1 year and 3 years were better after LSG than GB. GERD was more frequent at 1 year after LSG and at 3 years after GB. Overall, available studies confirmed that GB and especially LSG, have worse prognosis for GERD than RYGB. In a non-RCT between RYGB and LSG,^64^ the resolution of GERD was significantly higher in RYGB (62.8%) compared to LSG (15.9%). Among the LSG cohort, the presence of preoperative GERD was associated with increased postoperative complications, gastrointestinal adverse events, and an increased need for revisional surgery (*P <* 0.05 for all). On the other hand, there is a lack of articles exclusively investigating silent GERD in patients undergoing bariatric interventions.^15^^,^^49^ In one study, a risk factor analysis for GERD after 1 year from bariatric surgery identified LSG and preoperative reflux esophagitis (Los Angeles grades B, C, D) as independent risk factors.^15^ The other study investigated the risk of postoperative GERD and showed that 66% (37/54 patients) of patients with preoperative silent GERD, defined as esophagitis grade (Los Angeles) B, C, D, and/or abnormal esophageal acid exposure in absence of symptoms, became symptomatic after LSG.^49^ Considering that grade A esophagitis was not included in these studies and GERD is not the only determinant of treatment strategy, changing surgical procedure only by the existence of mild esophagitis is not reasonable. We therefore conclude that LSG is not recommended for patients with severe silent GERD.

9. **a) Post-operative EGDS should be performed after bariatric surgery in case of de-novo upper gastrointestinal symptoms **  **b) Post-operative EGDS should be performed within 5 years from LSG to rule out Barrett’s esophagus.**

Agree: 100% [D + (0%); D (0%); D− (0%); A− (0%); A (27.3%); A + (72.7%)].

*Conditional* Recommendation—Very low quality evidence

**Summary of evidence:**

Several nonrandomized retrospective studies investigated EGDS findings following RYGB, LSG, or GB.^65–73^ In one study, based on the data of 110 patients undergoing RYGB, compared to the pre-operative endoscopy, there was a significant decrease in postoperative prevalence of HH, esophagitis, and gastritis, compared to the preoperative endoscopy (*P* < 0.001 for all), while the most frequently reported surgical complications were stenosis of the gastroenteric anastomosis (35.5%) and formation of marginal ulcers (8.2%).^67^ In another retrospective study that enrolled 42 patients undergoing RYGB, compared to pre-operative endoscopy, the prevalence of esophagitis changed from 16.7% to 15.4% and the prevalence of gastritis decreased from 45.2% to 21.2%. In contrast, the prevalence of gastric or gastrojejunal ulcers increased from 4.8% to 9.6%.^68^ Of note, it has been recently shown that patients who underwent conversion from LSG to RYGB had a significantly higher incidence of marginal ulcers when compared to those who underwent RYGB or conversion from GB to RYGB.^74^ With regards to LSG, evidence on the prevalence of postoperative esophagitis is conflicting, with retrospective studies showing either an increase or a decrease in the prevalence of esophagitis.^69^^,^^70^ However, worsening of esophageal mucosal damage and conversion to another surgery are more common following LSG.^59^^,^^64^ In addition, studies on the de novo incidence of Barrett’s esophagus in patients undergoing LSG have reported incidence rates of up to 18.8%, with most patients remaining asymptomatic.^71–73^ Accordingly, routine endoscopic follow-up seems reasonable in this group of patients. More limited follow-up data are available for GB to date. In a retrospective study including 18 patients with preoperative and postoperative upper endoscopy, the prevalence of esophagitis increased from 16.7% to 30% after a mean of 30.1 months (range, 5–67) following GB. In another retrospective study based on the data of 23 patients undergoing GB, the prevalence of preoperative versus postoperative esophagitis was increased (61.5% vs. 69.5%), and 39.1% of patients had developed a postoperative pouch 6 months following surgery.^66^

Thus, according to the limited data available, postoperative EGDS should be performed after bariatric surgery in case of *de novo* upper gastrointestinal symptoms to identify potential mucosal injuries. The panel thought it reasonable to perform EGDS in all cases within 5 years following bariatric surgery to rule out Barrett’s esophagus.

10. **We recommend PPI use in the postoperative management of patients undergoing gastric bypass or biliopancreatic diversion for the prophylaxis of marginal ulcers.**

Agree: 81.8% [D + (9.1%); D (0%); D− (0%); A− (9.1%); A (45.4%); A + (36.4%)].

*Conditional Recommendation—Very low quality evidence*

**Summary of evidence:**

Proton pump inhibitors (PPIs) are usually prescribed before and after bariatric procedures, but there is lack of conclusive data regarding duration and dosage of the treatment, leaving the decision to the experience of individual centers.^13^ PPIs are typically prescribed to avoid the formation of marginal ulcers, a complication reported in up to 16% of patients following bariatric procedures.^75^ However, PPIs can also be prescribed before surgery either for GERD/dyspeptic symptoms, or for HP eradication.^76^ In a single-center retrospective study including 568 patients undergoing RYGB, marginal ulcers were diagnosed in 15.1% of patients, at a median time of 14.2 months after surgery. Of note, a 4- to 6-week PPI course (dexlansoprazole 60 mg, esomeprazole 40 mg, or pantoprazole 40 mg daily) before surgery decreased the risk of marginal ulcers with a hazard ratio of 0.54. In contrast, preexisting long-lasting PPI therapy did not significantly influence the development of marginal ulcers.^13^ A systematic review with meta-analysis investigated the utility of postsurgery PPI therapy in a cohort of 2114 patients from seven studies. Compared to patients taking PPIs (omeprazole 20 mg or esomeprazole 20 mg daily for up to 90 and 60 days, respectively), controls not taking PPIs developed twice as many marginal ulcers.^75^ In a single-center study including 610 patients undergoing RYGB, a lower proportion of patients receiving postoperative pantoprazole 40 mg developed marginal ulcers compared to controls (1.2% vs. 7.3%, *P* < 0.001).^77^ Similarly, patients using PPIs experienced fewer gastro-intestinal complaints (*P* < 0.001). Kang et al. evaluated data from 1016 patients undergoing RYGB who were administered a 30- or 90-day PPI (lansoprazole 30 mg daily) course after surgery. The incidence of marginal ulcers was significantly lower in those receiving a longer course of therapy (6.5% vs. 12.4%, *P* < 0.05).^78^ In another study, the impact of different modality of PPI assumption (open capsules [OC] or intact capsules [IC]; including omeprazole, pantoprazole, or esomeprazole 20 mg or 40 mg twice daily or lansoprazole 30 mg twice daily) was evaluated in terms of effectiveness in marginal ulcers healing. Data from a cohort of 162 patients showed that the median time for ulcer healing was lower in those treated with OC compared to IC (91 vs. 342 days, *P* < 0.001). Interestingly, OC resulted in the only independent predictor of time to ulcer healing (*P* < 0.001).^79^

In conclusion, available evidence suggests that postprocedural PPI therapy could be effective in reducing the risk of marginal ulcers development and promote healing. Further prospective and controlled studies are required to strengthen this recommendation and to define the dosage and duration of PPI treatment. Concerning preprocedural PPI therapy, apart from specific and well-defined indications (e.g. HP eradication, erosive esophagitis), available data are scarce and conflicting.

## LITERATURE GAPS REQUIRING ADDITIONAL STUDIES

Most published studies investigating the topic of these guidelines have several limitations (i.e. retrospective, case series, uncontrolled, etc.) that reduce the quality of evidence. We believe that efforts should be put in place to increase the level of evidence regarding esophago-gastric disorders affecting obese patients who are candidates for bariatric procedures.

Future studies should have a prospective design and should address the impact of accurately evaluating GERD and esophageal motor function in the selection of one type of bariatric intervention compared to another. In addition, high-quality studies investigating GERD outcomes across the various bariatric procedures in the long-term are also needed, overcoming methodological flaws of available literature. In addition, well-designed studies investigating the outcomes of endoscopic bariatric procedures are needed. Table 2 provides a list of key aspects that require further investigation and new research areas that need exploration.

**Table 2:** Research needs regarding GERD workup in patients undergoing bariatric procedures

**Area of interest**	**Research need**
HHR	There is unclear evidence that HHR improves pre-existing GERD symptoms or reduces *de novo* onset of GERD There is a need for evidence supporting that HHR alongside bariatric procedures improves post-procedural outcomes
Atypical GERD	There are no prospective studies that evaluated the impact of reflux assessment before bariatric procedures in patients with atypical symptoms. Similarly, the occurrence of atypical symptoms after bariatric interventions and associated reflux-monitoring findings has never been systematically assessed
Silent GERD	It is unclear whether silent GERD should be investigated in all patients undergoing bariatric interventions It is unclear whether silent GERD should influence the choice of the bariatric procedure to perform
HRM	Epidemiological data on pre- and post-bariatric procedures esophageal motor disorders according to the latest Chicago Classification (version 4.0) are needed It is unknown the impact of pre-existing motor disorders in both the decision-making process about the type of procedure and outcomes of the different interventions Clinical relevance and mid- long-term evolution of POSED is unclear
Post-operative EGDS	No high-quality data on *de novo* esophagitis incidence after LSG are available No high-quality data on the interval between bariatric procedures and the first follow-up EGDS for the screening of Barrett’s esophagus are available
PPI therapy	There is a need for prospective, randomized, controlled trials regarding the impact of preoperative and postoperative PPI treatment for the prophylaxis of marginal ulcers It is unclear if a prophylactic PPI treatment, before/after the bariatric intervention, may influence the development of *de novo* gastrointestinal complains
HP screening	There is a need for prospective, randomized, controlled trials regarding the impact of the systematic HP screening and eradication both in terms of outcomes of bariatric procedures and the development of *de novo* gastrointestinal problems
Endoscopic bariatric procedures	There is scarce data concerning the selection of candidates for and outcomes of endoscopic bariatric procedures according to GERD status *De novo* esophago-gastric comorbidity including GERD, and long-term outcomes of endoscopic bariatric procedures should be evaluated

GERD, gastro-esophageal reflux disease; HHR, hiatal hernia repair; HRM, high-resolution manometry; POSED, postobesity surgery esophageal dysfunction; EGDS, esophagogastroduodenoscopy; LSG, laparoscopic sleeve gastrectomy; HP, *Helicobacter pylori*.

## CONCLUSION

Obesity is a highly prevalent disease that affects up to 22.4% of the population in the Americas and up to 20% in Europe, accounting for around 650 million obese adults worldwide.^80^ In addition, a BMI 30–40 kg/m^2^ is associated with almost 50%, and a BMI over 40 kg/m^2^ is associated with 100%, greater healthcare costs due to management of obesity-related comorbidities.^81^ Bariatric surgery represents the most effective intervention to treat severe obesity and improve both morbidity and mortality. However, it is important to note that obese patients often suffer from gastrointestinal conditions including GERD and HH, and bariatric procedures may further deteriorate esophageal motor abnormalities. Accordingly, an accurate, patient-tailored, preoperative assessment of gastro-esophageal function is important in the setting of bariatric surgical practice.

This consensus, endorsed by the ISDE and ESDE, provided evidence and experience-based recommendations on the most appropriate diagnostic workup for the gastro-esophageal assessment in patients undergoing bariatric interventions. An international, multidisciplinary group of experts summarized key aspects in ten statements relevant to the management of bariatric patients. This document is intended to provide a practical guideline to support clinicians in their decision-making process, improve the outcomes of patients undergoing bariatric interventions, and reduce complications.

## ABBREVIATIONS

AET, acid exposure time; ARM, ambulatory reflux monitoring; BE, barium esophagogram; BMI, body mass index; DMS, DeMeester Score; EB, endoscopic balloon placement; EGDS, esophagogastroduodenoscopy; GB, gastric banding; GERD, gastroesophageal reflux disease; HHR, HH repair; HRM, high-resolution esophageal manometry; IEM, ineffective esophageal motility; ISDE, International Society for Diseases of the Esophagus; LES, lower esophageal sphincter; LSG, laparoscopic sleeve gastrectomy; OR, odds ratio; PICO, population, intervention, comparator, and outcome; PPI, proton pump inhibitor; QoL, quality of life; RYGB, Roux-en-Y gastric by-pass

## DATA SHARING STATEMENT

No additional data are available.

## Notes

**Specific author contributions**: All authors were involved in planning, collecting data, writing of the manuscript, and approval of the final version of the manuscript.

## Notes

**Financial support**: None.

## Notes

**Conflicts of interest**: Pierfrancesco Visaggi, Brigida Barberio, Ryu Ishihara, Matteo Ghisa, Philip Chiu, Ryu Ishihara, Goeffrey P Kohn, and Ian Wong do not report conflict of interest. Sergey Morozov has served as speaker for AstraZeneca and Takeda; he received research support from Russian Science Foundation. Sarah K Thompson: Consultant for Ferronova; Cesare Hassan: Medtronic (equipment loan); Fujifilm (consulting); Olympus (consulting; NEC (equipment loan); Satisfy (equipment loan); Odin (equipment loan); AIM (equipment loan). Edoardo Vincenzo Savarino has served as speaker for Abbvie, Agave, AGPharma, Alfasigma, Aurora Pharma, CaDiGroup, Celltrion, Dr Falk, EG Stada Group, Fenix Pharma, Fresenius Kabi, Galapagos, Janssen, JB Pharmaceuticals, Innovamedica/Adacyte, Malesci, Mayoly Biohealth, Omega Pharma, Pfizer, Reckitt Benckiser, Sandoz, SILA, Sofar, Takeda, Tillots, Unifarco; has served as consultant for Abbvie, Agave, Alfasigma, Biogen, Bristol-Myers Squibb, Celltrion, Diadema Farmaceutici, Dr. Falk, Fenix Pharma, Fresenius Kabi, Janssen, JB Pharmaceuticals, Merck & Co, Nestlè, Reckitt Benckiser, Regeneron, Sanofi, SILA, Sofar, Synformulas GmbH, Takeda, Unifarco; he received research support from Pfizer, Reckitt Benckiser, SILA, Sofar, Unifarco, Zeta Farmaceutici.
